# Universal home visits improve male knowledge and attitudes about maternal and child health in Bauchi State, Nigeria: Secondary outcome analysis of a stepped wedge cluster randomised controlled trial

**DOI:** 10.7189/jogh.12.04003

**Published:** 2022-02-05

**Authors:** Anne Cockcroft, Khalid Omer, Yagana Gidado, Muhd Chadi Baba, Amar Aziz, Umaira Ansari, Adamu Ibrahim Gamawa, Rilwanu Mohammed, Salisu Abubakar Galda, Neil Andersson

**Affiliations:** 1 CIET-PRAM, Department of Family Medicine, McGill University, Montreal, Canada; 2 Centro de Investigácion de Enfermedades Tropicales, Universidad Autónoma de Guerrero, Acapulco, Guerrero, Mexico; 3 Federation of Muslim Women’s Associations of Nigeria (FOMWAN), Bauchi, Nigeria; 4 Bauchi State Primary Health Care Development Agency, Bauchi, Nigeria; 5 Toro Local Government Health Authority, Bauchi State, Nigeria

## Abstract

**Background:**

The World Health Organization recommends increased male involvement to improve maternal and newborn health in low- and middle-income countries, but few studies have measured the impact of male-engagement interventions on targeted men. A trial of universal home visits to pregnant women and their spouses in Nigeria improved maternal and child health outcomes. This analysis examines the impact of the visits on male spouses.

**Methods:**

In Toro Local Government Area in Bauchi State, Nigeria, we randomly allocated eight wards into four waves, beginning the intervention at one-year intervals. The intervention comprised two-monthly evidence-based home visits to discuss local risk factors for maternal and child health with all pregnant women and their male spouses. Measured secondary outcomes of the intervention in the men included knowledge about danger signs in pregnancy and childbirth, beliefs about heavy work in pregnancy, discussion with their wives about pregnancy and childbirth, knowledge about causes and intentions about management of childhood diarrhoea, and views about childhood immunisation. The analysis compared outcomes between men in visited wards (intervention group) and pre-intervention wards (control group), using a cluster *t* test. Generalised linear mixed modelling accounted for the effect of socio-economic differences on the measured impact.

**Results:**

The analysis included 6931 men in the intervention group and 9434 in the control group. More men in the intervention group knew four or more danger signs in pregnancy (risk difference (RD) = 0.186, 95% confidence interval (CI)  = 0.044 to 0.327), and three danger signs in childbirth (RD = 0.091, 95%CI = 0.013 to 0.170), thought pregnant women should reduce heavy work before the third trimester (RD = 0.088, 95% CI = 0.015 to 0.162), and had discussed pregnancy and childbirth with their spouse (RD = 0.157, 95% CI = 0.026 to 0.288). More knew correct management of childhood diarrhoea with fluids and feeding (RD = 0.300, 95% CI = 0.203 to 0.397) and less would give a child medicine to stop diarrhoea (RD = 0.206, 95% CI = 0.125 to 0.287). Socio-economic differences did not explain the effect of the intervention on any of the outcomes.

**Conclusion:**

Universal home visits improved knowledge of male spouses about maternal and child health, which could contribute to improved maternal and child outcomes.

**Trial registration:**

ISRCTN, ISRCTN82954580. 11 August 2017. Retrospectively registered. http://www.isrctn.com/ISRCTN82954580

Maternal and child mortality remain high in many parts of the world [1,2]. The World Health Organization identifies male involvement in reproductive health as a priority for improving maternal and newborn health [3]. Several systematic reviews of studies in low- and middle-income countries conclude that male involvement is associated with increased use of maternal care services, such as attendance of women for antenatal care, institutional delivery, skilled birth attendance, and post-natal visits [4-6]. The impact of interventions to increase male involvement on maternal and newborn morbidity and mortality is not clear [4,5]. There are concerns that in some contexts efforts to promote male involvement in reproductive health may cause harm, reinforcing gender stereotypes and increasing gender inequality and male dominance [4,7]. Some studies have measured male behaviour after interventions indirectly by asking women about actions of their partners [8,9]. Few studies of interventions to engage men in maternal and newborn health have directly measured outcomes of knowledge or behaviour among men [10-13].

A recent stepped wedge cluster randomized controlled trial of home visits to pregnant women and their spouses in Bauchi State, Nigeria [14] reported large and significant impacts on maternal health outcomes [15] and on child health outcomes [16]. The visits targeted upstream actionable risk factors for maternal health, based on the findings from a previous survey in the state [17].

The conceptual framework underlying the universal home visits to pregnant women and their spouses explicitly recognised male involvement as crucial for household actions to improve maternal and child health. We hypothesized that visiting male spouses would improve their knowledge and attitudes and encourage them to act in support of maternal and child health. The analysis reported here has two objectives. First, we examined the impact of the home visits on male knowledge and attitudes, comparing visited wards (intervention group) with pre-visited wards (control group) in the stepped-wedge design. Second, within the intervention group, we examined changes in male knowledge and beliefs over the course of two or more visits, to look for associations between positive changes and characteristics of the men.

## METHODS

### Setting

Bauchi State in North Eastern Nigeria has a population of about 5 million based on projections from the 2006 census. Over 95% of the population are Muslim and polygamy is common. Some 63% of women and 44% of men in Bauchi have no formal education, compared with 35% and 22% nationally [18]. National maternal mortality in Nigeria is among the highest in the world [1], and rates are even higher in Bauchi [19]. Child immunization rates (children aged 12-23 months who received all basic vaccinations) are lower in Bauchi (19.6%) than nationally (31.3%), and two week diarrhoea prevalence in children under 5 years old is higher in Bauchi (34.1%) than nationally (12.8%) [18]. Toro is the largest Local Government Area (LGA) in Bauchi State, with a projected population of 487 100 in 18 wards (smallest administrative units). The research findings are specific to this setting but could be relevant to other low resource settings.

### Design of the trial

**Figure 1:**
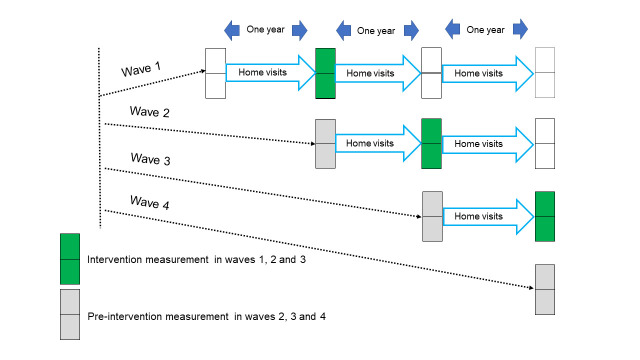
Stepped wedge design of the trial. Each square box represents a ward. Each wave comprised two wards. The intervention measurement comprises measurements accumulated during one year of home visits in waves 1, 2 and 3. The pre-intervention measurement comprises the measurements made on baseline visits to all households in waves 2, 3 and 4.

The published trial protocol describes the overall trial design and methods in detail [14]. The trial took place in eight of the 18 wards in Toro LGA. We randomly allocated these wards into four waves of two wards each. **Figure 1** shows the stepped-wedge design of the overall trial. Successive waves of two wards began the home visits intervention at yearly intervals. The intervention group comprises waves 1, 2 and 3 wards after one year of visits and the control (pre-intervention) group comprises waves 2, 3 and 4 wards at their baseline. We did not undertake a separate baseline measurement in wave 1 wards and wave 4 wards did not go on to receive visits within the timescale of the project. The home visits began in March 2016 in wave 1. Visits in all waves and collection of baseline information in wave 4 finished by the end of December 2019. Logistic and financial constraints precluded extension of home visits into wave 4 wards after 2019.

### Participants

All households in the eight wards participated in the study. The visits were universal by design; an initial mapping in collaboration with community leaders identified all households in each community and all were eligible for visits. Female home visitors visited every household every two months and, on each visit, identified any new pregnancies. No households refused the visits, which were strongly endorsed by community leaders. Home visitors collected baseline socio-demographic characteristics of the households at the pre-intervention visits in wards in waves 2, 3 and 4 and in the first intervention visit in wave 1 wards. For the analysis reported here, the intervention group comprised spouses of pregnant women in the visited wards who were visited by a male home visitor at least once during the pregnancy and who provided data about their knowledge and attitudes in a visit soon after the birth of the child (total of at least two visits). The control group comprised spouses of women giving birth within the last 12 months or with a child aged 12-18 months at the baseline visit in the pre-intervention wards.

### Intervention

The trial protocol describes the intervention in detail [14]. The intervention consisted of two-monthly home visits to all pregnant women and their spouses, with a further visit after the birth and a final visit when the child was aged 12-18 months old. Female home visitors visited and interviewed the women and male home visitors separately visited and interviewed their spouses. Most of the home visitors worked within their own home communities. They received training over 11 days, including classroom sessions and field practice. We selected only trainees who attained the required skills to carry out the home visits. Each team of one female and one male home visitor covered a catchment area of about 300 households. The wards had from 8 to 27 catchment areas to cover all the households. The female home visitors visited every household in their catchment areas every two months during daylight hours. In the first visit they registered and collected demographic and socio-economic information from each household, listing all women of childbearing age (14-49 years). During subsequent two-monthly visits the female home visitors checked with the women of childbearing age if any of them had become pregnant since the last visit. They registered and followed all pregnant women with two-monthly visits during the pregnancy and in a visit after the birth. The male home visitors visited the spouses of the pregnant women, also approximately two-monthly, often in the evening or on weekends. The home visitors interacted with the women and their spouses using a surveillance questionnaire and the same structured discussion guide. The discussion guide included the four risk factors for maternal health identified in a survey in Bauchi State (heavy work in pregnancy, domestic violence in pregnancy, lack of knowledge about danger signs, and lack of discussion with the spouse) [17]. The guide also covered early childhood health and care, especially prevention and management of diarrhoea and routine immunisation, again using evidence from a survey in Bauchi State [20]. As described in the protocol [14], in one of each pair of wards, randomly allocated, the home visitors supplemented their evidence-based discussions with short video clips about the four risk factors for maternal health. A paper examining the added value of the video clips on maternal, child and male outcomes is in preparation.

### Outcomes

The key outcomes among male spouses were knowledge and attitudes relevant to the targeted maternal and child health outcomes. They included: knowledge of danger signs during pregnancy and childbirth; belief that a pregnant woman should reduce heavy work; discussion with their spouse about pregnancy and childbirth; knowledge of poor hygiene as a cause of childhood diarrhoea; knowledge about giving extra fluid and continued feeding for management of childhood diarrhoea; intention not to give anti-diarrhoea medicines to young children; perception of childhood immunization as worthwhile; and discussion with their spouse about immunization of children. To compare between men in the intervention group and men in the control group, we measured these outcomes on the latest visit prior to the one-year cut-off among visited men and in the baseline visit to households among control (pre-intervention) men.

### Sample size

We calculated the trial sample size based on maternal outcomes [14]. Since male outcomes (knowledge and attitudes) were more common than the measured maternal outcomes, the trial had more power to detect significant differences in these outcomes.

### Randomization and masking

We selected eight out of 18 wards in Toro LGA as eligible for participation in the trial, excluding those where the security situation was too precarious. We divided these eight wards into two sets, geographically apart, and randomly selected four wards from each of these two sets, generating four pairs of wards. An epidemiologist not involved in the fieldwork (NA) randomly assigned the pairs of wards into four waves for implementation, using a computer-generated random sequence.

The home visitors could not be unaware of the allocation of the intervention. We used standardised procedures to administer questionnaires and record responses in pre-intervention and intervention visits and the questionnaire used in both contexts was the same. The home visitors had no reason to conduct the process differently in intervention and pre-intervention visits. The households gave their consent to receive visits and to respond to questionnaires, but they were not aware that they were allocated to a treatment or control group.

### Statistical methods

Analysis relied on CIETmap open-source software [21], which provides a user-friendly interface with the R statistical language. We used the Mantel-Haenszel procedure [22] to examine differences in socio-demographic characteristics between the intervention and control groups at baseline, reporting the odds ratio (OR) and 95% confidence interval (CI). The socio-demographic characteristics included age (30 years or less vs older), marital status (one wife vs more than one), whether the household had enough food in the last week (as a measure of poverty), education (any formal education or not), occupation (better paying occupation or not), and urban or rural residence.

To assess the impact of home visits on male outcomes we used a cluster *t* test, comparing outcomes between men in intervention and control groups. We report the risk difference (RD), relative risk reduction (RRR) and 95% confidence intervals (CI) of these parameters. To examine whether differences in socio-economic characteristics could explain the intervention effect, we used generalized linear mixed modelling (GLMM) [23]. We included ward and catchment area (each area of about 300 households) as random effect variables to allow for clustering at these two levels. We first ran the model including only the intervention variable, then repeated it with an initial saturated model including socio-economic characteristics in the intervention and control groups, stepping down to a model where all variables were significantly associated with the outcome. We report the OR and 95% confidence intervals.

A supplementary analysis examined changes in male knowledge and beliefs between their first and last visit, categorising these as negative to positive, negative to negative, positive to positive, and positive to negative. Among the subset with negative knowledge or beliefs on the first occasion, we compared those who shifted to positive with those who remained negative, and looked for associations between this “positive change” and baseline socio-economic characteristics of the men in a multivariate analysis [22], allowing for clustering by catchment area [24].

### Ethical considerations

We followed the principles embodied in the Declaration of Helsinki to conduct the trial. We shared the study protocol and design in non-technical terms with the community, ward and LGA leadership and sought their approval before starting the trial. The home visitors sought informed consent from each household, woman and spouse to be visited and to respond to questionnaires. All responses recorded from participants were confidential and no names or identifying information were linked to the recorded data reaching the server.

## RESULTS

### Participants’ flow

**Figure 2:**
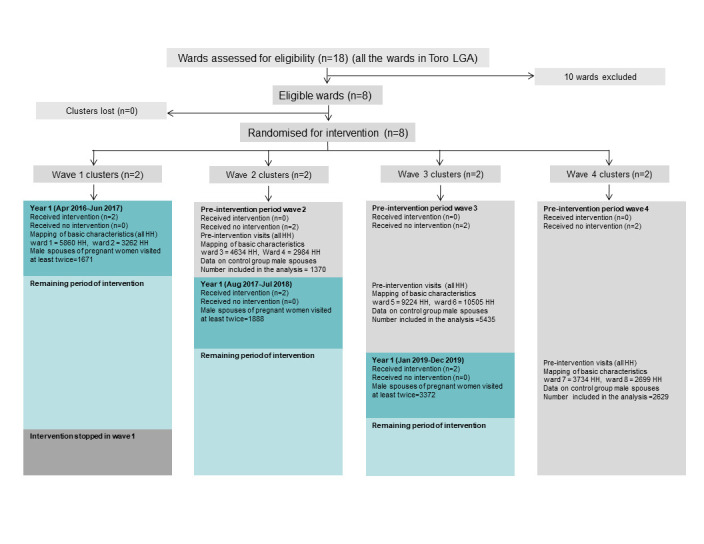
Trial flowchart by allocated sequence and period. Dark blue blocks are the first year of the periods of intervention. Light blue blocks are the periods of intervention after the first year (not included in the analysis). Grey blocks are the pre-intervention periods in waves 2, 3 and 4. H – household.

**Figure 2** shows the flow of wards and participants into the intervention and pre-intervention (control) groups for each wave during the trial.

### Recruitment and number analysed

**Table 1** shows the implementation schedule of the intervention (home visits) in waves 1-3 and the cut-off dates for the first year of intervention in each wave. The intervention began at about one- year intervals across the waves, and in waves 2 and 3 began with a baseline survey.

**Table 1:** Intervention implementation schedule

	Wave 1	Wave 2	Wave 3
Start date	March-April 2016	July-August 2017	January 2019
Cut off for first year of intervention	June 2017	July 2018	December 2019

Data on male knowledge and attitudes were available among 16 365 men. In the intervention group these were 6931 male spouses of pregnant women visited during their pregnancies. In the control group these were 9434 male spouses of women who gave birth within the last 12 months or who had a child aged 12-18 months old. **Table 2** shows the trial flow of male participants. The number of men in the intervention and control groups from wave 3 reflects the large population in the two wards comprising wave 3.

**Table 2:** Trial flow of male participants

	Wave 1	Wave 2	Wave 3	Wave 4	Total
**Intervention group:**					
Visited at least once	2723	2610	6143	N/A	11 476
Not eligible for follow-up within cut-off period*	0	0	2168	N/A	2168
Lost to follow-up†	1052	722	603	N/A	2377
Number for analysis in intervention group‡	1671	1888	3372	N/A	6931
**Control group (Pre-intervention)**	N/A	1370	5435	2629	9434

*These were men visited for the first time towards the end of the intervention period and not scheduled for a follow-up within the intervention period.

†These were men who did not receive any visit after the first visit, although they should have been due to receive at least one more visit.

‡The men included in the analysis in the intervention group all had at least two visits.

### Socio-economic characteristics

**Table 3** shows socio-economic characteristics of the men in the intervention and control groups. The intervention group were men from waves 1, 2 and 3 after one year of intervention and the control group were men in waves 2, 3 and 4 at baseline (pre-intervention). Men in the intervention group were slightly older, with a lower proportion aged 30 years or less, and more of them had some formal education and a better income occupation. About a third of the men in the intervention group were from urban communities, compared with half of the men in the control group.

**Table 3:** Socio-economic characteristics of men included in the analysis

Characteristic	% (fraction)		OR (95% CI)
	**Intervention group**	**Control group**	
Total number of participants	6931	9434	
Mean age (years)	38 (6446) (SD = 8)	37 (9434) (SD = 9)	
Aged 30 y or less	20.5 (1319/6446)	27.6 (2604/9434)	0.67 (0.62-0.73)
With only one wife	63.8 (4423/6931)	67.4 (6363/9434)	0.85 (0.80-0.91)
With some formal education	74.5 (4792/6431)	58.5 (5502/9407)	2.08 (1.94-2.23)
With a better income occupation	51.7 (3329/6435)	45.4 (4278/9428)	1.29 (1.21-1.38)
From female headed households	0.3 (19/6693)	0.3 (29/9210)	0.90 (0.45-1.65)
From household with enough food in last week	96.2 (6424/6681)	97.5 (8970/9197)	0.63 (0.52-0.76)
From urban communities	36.6 (2536/6931)	51.7 (4878/9434)	0.54 (0.51-0.57)

OR – odds ratio; 95% CI – 95% confidence interval

### Outcomes and estimation of impact

**Table 4** shows knowledge and attitudes of men related to maternal and child health outcomes, with the results of the cluster *t* test. Men in the intervention group had better knowledge about danger signs during pregnancy and childbirth than men in the control group. This was mainly apparent for knowing more than one danger sign, with significantly higher proportions of men in the intervention group knowing three or more danger signs in pregnancy and childbirth. In both the intervention and control groups, nearly all men said that women should reduce heavy work at some point during pregnancy. The proportion who thought women should reduce heavy work before the third trimester of pregnancy was significantly higher in the intervention group. Significantly higher proportions in the intervention group said they had discussed pregnancy and childbirth with their spouses or had discussed these issues with them often.

**Table 4:** Knowledge and attitudes related to maternal and child health among 6931 men in the intervention group and 9434 men in the control group*

Outcome	Proportion (number)		RRR (95%CIca)	RD (95%CIca)	NNT	ICC
	Intervention group	Control group				
**Related to maternal health outcomes:**						
Know any danger sign during pregnancy	0.932 (6458 / 6931)	0.872(8227 / 9434)	0.064 (-0.007 to 0.136)	0.060 (-0.008 to 0.128)		0.032
Know 3 or more danger signs during pregnancy	0.409 (2833 / 6931)	0.228(2154 / 9434)	**0.441(0.076 to 0.807)**	0.264(-0.009 to 0.370)		0.108
Know 4 or more danger signs during pregnancy	0.261 (1811 / 6931)	0.076 (715 / 9434)	**0.710 (0.391 to 1.029)**	**0.186 (0.044 to 0.327)**	6	0.097
Know any danger sign during childbirth	0.816 (5656 / 6931)	0.745 (7029 / 9434)	0.087(-0 · 066 to 0.240)	0.071 (-0 · 058 to 0.200)		0.061
Know 3 danger signs during childbirth	0.146(1013 / 6931)	0.055(517 / 9434)	**0.625(0.282 to 0.968)**	**0.091(0.013 to 0.170)**	11	0.044
Think women should reduce heavy work in pregnancy	0.949 (6546 / 6931)	0.933 (8799 / 9434)	0.017(-0.019 to 0.054)	0.016(-0.019 to 0.051)		0.013
Think women should reduce heavy work before 3rd trimester	0.798(5507 / 6897)	0.710(6698 / 9434)	**0.111(0.023 to 0.199)**	**0.088(0.015 to 0.162)**	12	0.018
Discussed pregnancy & childbirth with spouse	0.965(6656 / 6897)	0.808(7623 / 9434)	**0.163(0.030 to 0.295)**	**0.157(0.026 to 0.288)**	7	0.110
Often discussed pregnancy & childbirth with spouse	0.877(6047 / 6897)	0.691(6522 / 9434)	**0.211(0.033 to 0.390)**	**0.185(0.019 to 0.352)**	6	0.106
**Related to child health outcomes:**						
Mention poor hygiene as cause of childhood diarrhoea	0.758(4734 / 6246)	0.650(6133 / 9434)	0.142 (-0.025 to 0.310)	0.108 (-0.027 to 0.243)		0.054
Know to give child with diarrhoea more fluids & continued feeding	0.504(3149 / 6246)	0.204(1923 / 9434)	**0.596(0.460 to 0.732)**	**0.300(0.203 to 0.397)**	4	0.027
Would *not* give child with diarrhoea medicine to stop the diarrhoea	0.223(1393 / 6246)	0.017(162 / 9434)	**0.923 (0.809 to 1.037)**	**0.206 (0.125 to 0.287)**	5	0.042
Think it is worthwhile to immunize children	0.981(6126 / 6246)	0.981(9259 / 9434)	0.001 (-0.019 to 0.017)	-0.001 (-0.018 to 0.017)		0.010
Discussed child immunization with spouse/family	0.940(5869 / 6246)	0.929(8766 / 9434)	0.011 (-0.069 to 0.091)	0.010 (-0.065 to 0.086)		0.057

RRR – relative risk reduction (1-RR (relative risk)), RD – risk difference, NNT – number needed to treat (1/RD), 95% CIca – cluster-adjusted 95% confidence interval, ICC – intra-cluster correlation

*Bold font indicates the contrast is significant at the 5% level

Some 11% more men in the intervention group mentioned poor hygiene around the household as a cause of childhood diarrhoea, but this difference was not statistically significant in the cluster *t* test. Beliefs about management of childhood diarrhoea were significantly better in the intervention group than in the control group: 30% more thought that children with diarrhoea should be given extra fluids and continued feeding and 21% more said they would not give a child medicine to stop diarrhoea. The perception of childhood immunization being worthwhile was nearly universal in both intervention and control groups, and nearly all in both groups said they had discussed childhood immunization with their family or spouse.

Further analysis examined whether baseline socio-economic differences between the intervention and control groups could explain the impact of the intervention on men’s knowledge and attitudes. The results of GLMM analysis for the effect of the intervention using a model including the intervention alone, and a model including the baseline socio-economic characteristics are shown in Table S1 in the **Online Supplementary Document**. Baseline socio-economic differences did not explain the effect of the intervention on any of the male outcomes.

### Changes in knowledge and attitudes among visited men

Knowledge and beliefs among visited men shifted between first and last visits (Table S2 in the **Online Supplementary Document**). As expected, positive changes were more prominent for the knowledge or attitudes that were less favourable at the first visit. For example, most men (88%, 6104/6931) knew at least one danger sign in pregnancy at the first visit; there were only 827/6931 (11.9%) without this knowledge and susceptible to a positive change. Less men knew at least three danger signs in pregnancy at the first visit (31.3%, 2171/6931) and more were susceptible to a positive change (68.7%, 4760/6931).

Multivariate analysis examined associations between characteristics of the men and positive change in knowledge or attitudes, among those men susceptible to positive change (Table S3 in the **Online Supplementary Document**). For most of the knowledge items, there were few or no associations between basic characteristics of the visited men and the likelihood of a positive change in their knowledge or beliefs. The analysis suggested that younger men and those less poor (based on food sufficiency) were more likely to increase their knowledge about danger signs. Younger men were less likely to increase their discussion with their spouses, while those with only one wife were more likely to increase their discussion with her. As shown in Table S2 in the **Online Supplementary Document**, few men on the first visit said they would not give a child with diarrhoea medicine to stop the diarrhoea (12.7%, 426/3366). This increased to 20.6% (692/3366) on the last visit. Younger men were more likely to change their view about giving medicines to stop diarrhoea. But more educated men were less likely to change their view about this after being visited.

## DISCUSSION

Male spouses who received evidence-driven home visits to discuss local risk factors for maternal and child health had significantly better knowledge and attitudes related to maternal and child health than did male spouses in the control group. Among visited men, the individual socio-economic characteristics we measured could not explain shifting from negative to positive knowledge and attitudes after two or more home visits.

In our study, home visitors shared with women and men (separately) the same local evidence about actionable upstream risk factors for maternal health: heavy work during pregnancy, domestic violence during pregnancy, lack of spousal discussion about pregnancy and childbirth, and lack of knowledge of danger signs during pregnancy and childbirth [17]. We previously reported that home visits led to marked reductions in these risk factors [15], all of which are actionable by men. This study confirms visited men were more likely to discuss these issues with their wives. We reported in other contexts that sharing information with stakeholders in communities – rather than simply telling them what to do – allows them to decide to take action to change their situation [25,26]. We hypothesized that sharing information with the male spouses of pregnant women would increase their knowledge and ability to make changes to support the health of their wives and newborn children; the findings we report here suggest this was indeed the case.

Our study expands the existing evidence base about the benefits of male involvement in reproductive health. The most commonly reported outcome of studies of male involvement is women’s use of maternal care services, such as antenatal care visits, skilled birth attendance, institutional delivery, and postnatal visits. Male involvement is itself often defined in terms of use of services. A recent systematic review of male involvement, defined as attendance to antenatal care with the partner, reported increased skilled birth attendance, increased institutional delivery and increased postpartum visits among women who attended antenatal care with a partner [27]. Less than half the included studies were randomised controlled trials, and study outcomes did not include maternal and child health indicators. Our trial showed an impact of the home visits on men’s knowledge and attitudes, as well as improvements in both maternal and child health outcomes [15,16].

Studies often assess the impact of interventions on men indirectly. Studies of community interventions in India and Pakistan assessed the impact on the participating men indirectly, through women’s reports about the behaviour of their partners, such as attendance at antenatal care [8,9]. Our study had the advantage of being able to measure directly the impact of the home visits on knowledge and behaviours among participating men. This is not the first study to make measurements directly among participating men. Previous studies have reported mixed results of different interventions targeting men and measuring the impact among the men. A study of a multi-media campaign targeting husbands in Indonesia reported that men exposed to the campaign gained new knowledge and took birth preparedness actions [10]. On the other hand, two studies, in South Africa and India, where men were invited to attend counselling sessions with their pregnant wives, did not find a significant increase in knowledge about danger signs among the men [11,12]. A more recent study in Tanzania, where community health workers visited homes and gave joint educational sessions to pregnant women and their spouses, reported that men in the intervention sites had increased knowledge of danger signs in pregnancy and childbirth, were more likely to accompany their wives to antenatal care and childbirth, and more likely to share decision-making about the place of delivery [13]. These results are consistent with our findings of increased knowledge about danger signs and increased discussion with the spouse about pregnancy and childbirth among men receiving the home visits.

In our study, only 23% of men in the control group knew three or more danger signs in pregnancy and only 5% knew three danger signs in childbirth. This accords with a qualitative study in northern Nigeria which described low levels of knowledge about danger signs among men [28]. Cross-sectional studies in Myanmar, Ethiopia and Bangladesh reported low levels of knowledge about pregnancy and childbirth danger signs among men and that men’s knowledge about maternal and newborn health was related to their practical involvement during pregnancy and childbirth [29-31]. It is encouraging that the home visits to men in our study led to improvements in knowledge and behaviours. However, we were not able to identify any factors associated with a greater likelihood of improvement from the visits. Future research could focus on understanding which men change as a result of the visits, and which do not; this could help to tailor the visits to maximise their impact.

### Strengths and limitations

Our study was a randomized controlled trial that measured outcomes in men directly, increasing our confidence that measured differences were due to the intervention. We demonstrated a significant impact on men in the context of a trial that also found significant impacts on maternal and child health outcomes, and not simply services utilization. Repeated measures of male knowledge and attitudes during the intervention allowed us to examine changes in outcomes over several visits and to explore the association between these changes and individual characteristics.

The trial had a small number of clusters (wards) in each group. We cannot exclude unmeasured imbalances between intervention and control clusters. This is less likely in the stepped-wedge design, where the wards in waves 2 and 3 were included in both their pre-intervention and intervention status.

All visited households had visits to both pregnant women and their male spouses, so we cannot tell if visiting the male spouses added value to the visits to pregnant women, or even if visiting male spouses could have led to changes in maternal and child outcomes in the absence of visits to pregnant women themselves. Future research could examine which elements of the home visits were most important in improving maternal, child and male outcomes and measure sustainability of the male knowledge changes associated with the visits.

## CONCLUSIONS

Evidence-driven home visits to the male spouses of pregnant women increased their knowledge about maternal, newborn and early child health and associated local risk factors and increased their discussion with their wives about these issues. Together with evidence of impact of the visits on maternal and child health outcomes, this supports the idea that community efforts to improve maternal and child health must involve men.

## Additional material


Online Supplementary Document
