# Supplementary material for: Universal home visits improve male knowledge and attitudes about maternal and child health in Bauchi State, Nigeria: Secondary outcome analysis of a stepped wedge cluster randomised controlled trial
Source: J Glob Health. 2022 Feb 5;12:04003. doi: 10.7189/jogh.12.04003 (PMC8818298; doi:10.7189/jogh.12.04003)
Supplement: Online Supplementary Document [file jogh-12-04003-s001.pdf]

Table S1. GLMM models for impact of the intervention (home visits) on outcomes of male knowledge and attitudes

Outcomes	Proportion (number)		OR (Robust 95% CI)	
	Intervention group	Control group	Intervention alone	Modelled with baseline characteristics
Related to maternal health outcomes				
Know any danger sign during pregnancy	0.932 (6458 / 6931)	0.872 (8227 / 9434)	**5.20 (2.25 – 11.98)**	**4.69 (2.04 – 10.82)**
Know 3 or more danger signs during pregnancy	0.409 (2833 / 6931)	0.228 (2154 / 9434)	2.69 (0.78 – 9.23)	2.67 (0.72 – 9.91)
Know 4 or more danger signs during pregnancy	0.261 (1811 / 6931)	0.076 (715 / 9434)	**5.55 (1.38 – 22.28)**	**5.78 (1.32 – 25.26)**
Know any danger sign during childbirth	0.816 (5656 / 6931)	0.745 (7029 / 9434)	**2.98 (1.43 – 6.22)**	**2.68 (1.19 – 6.06)**
Know 3 danger signs during childbirth	0.146 (1013 / 6931)	0.055 (517 / 9434)	**4.21 (1.48 – 11.97)**	**4.26 (1.49 – 12.20)**
Think women should reduce heavy work in pregnancy	0.949 (6546 / 6897)	0.933 (8799 / 9434)	**2.98 (1.58 – 5.60)**	**2.74 (1.40 – 5.34)**
Think women should reduce heavy work before 3rd trimester	0.798 (5507 / 6897)	0.710 (6698 / 9434)	**3.25 (1.91 – 5.56)**	**3.16 (1.83 – 5.44)**
Discussed pregnancy & childbirth with spouse	0.965 (6656 / 6897)	0.808 (7623 / 9434)	**4.48 (1.10 – 18.22)**	**6.46 (2.08 – 20.05)**
Often discussed pregnancy & childbirth with spouse	0.877 (6047 / 6897)	0.691 (6522 / 9434)	**7.49 (2.48 – 22.60)**	**4.91 (1.68 – 14.30)**
Related to child health outcomes				
Mention poor hygiene as cause of childhood diarrhoea	0.758 (4734 / 6246)	0.650 (6133 / 9434)	**3.04 (1.59 – 5.83)**	**2.95 (1.20 – 7.25)**
Know to give child with diarrhoea more fluids & continued feeding	0.504 (3149 / 6246)	0.204 (1923 / 9434)	**8.62 (4.03 – 18.43)**	**8.30 (4.35 – 15.83)**
Would *not* give child with diarrhoea medicine to stop the diarrhoea	0.223 (1393 / 6246)	0.170 (162 / 9434)	**25.04 (9.78 – 64.10)**	**22.81 (8.61 – 60.39)**
Think it is worthwhile to immunize children	0.981 (6126 / 6246)	0.981 9259 / 9434)	1.12 (0.52 – 2.41)	0.96 (0.49 – 1.92)
Discussed child immunization with spouse/family	0.940 (5869 / 6246)	0.929 (8766 / 9434)	2.56 (0.72 – 9.05)	1.92 (0.67 – 5.46)

Notes:

1. Both models include ward and catchment area as random effects variables

2. Some associations with the intervention are significant in the GLMM model including both ward and catchment area as random effects, that were not significant in the cluster t test (Table 5), which considered only ward level clustering

2. Baseline characteristics included in the initial saturated models: Type of community (urban vs rural and rural remote); education (any formal education vs none); occupation (better income vs poor income); number of wives (one vs more); Household food security (enough vs not enough food in last week); sex of the household head (female vs male); age (up to 30 years v/s older)

Table S2. Changes in male knowledge and attitudes among the 6931 men in visited wards that had at least two visits

Knowledge or belief	Percentage (number) that changed: From negative to positive	From negative to negative	From positive to positive	From positive to negative	Total number that began negative
Relevant to maternal health outcomes[1]					
Know any danger sign during pregnancy	9.2 (637)	2.7 (190)	84.0 (5821)	4.1 (283)	827
Know 3 or more danger signs during pregnancy	18.5 (1280)	50.2 (3480)	22.4 (1553)	8.9 (618)	4760
Know 4 or more danger signs during pregnancy	15.9 (1102)	69.7 (4828)	10.2 (709)	4.2 (292)	5930
Know any danger sign during childbirth	17.5 (1210)	10.9 (758)	64.1 (4446)	7.5 (517)	1968
Know 3 danger signs during childbirth	10.5 (730)	81.9 (5675)	4.1 (283)	3.5 (243)	6405
Think women should reduce heavy work in pregnancy	6.4 (438)	1.8 (126)	88.5 (6094)	3.3 (225)	564
Think women should reduce heavy work before 3rd trimester	14.7 (1013)	8.9 (616)	65.1 (4484)	11.2 (770)	1629
Ever discussed pregnancy & childbirth with pregnant spouse	8.6 (585)	1.3 (90)	88.0 (6001)	2.2 (147)	675
Often discussed pregnancy & childbirth with pregnant spouse	14.5 (988)	7.6 (521)	73.3 (4999)	4.6 (315)	1509
Relevant to child health outcomes[2]					
Mention poor hygiene as a cause of childhood diarrhoea	20.6 (699)	12.5 (422)	55.2 (1869)	11.7 (397)	1121
Know to give more fluid and continued feeding to child with diarrhoea	27.7 (862)	36.8 (1144)	23.1 (718)	12.4 (385)	2006
Would not give child with diarrhoea medicine to stop the diarrhoea	13.6 (458)	73.7 (2482)	7.0 (234)	5.7 (192)	2940
Think it is worthwhile to immunize children	2.8 (95)	0.2 (6)	95.4 (3211)	1.6 (54)	101
Discussed child immunization with wife and family	6.4 (216)	2.1 (72)	88.4 (2976)	3.0 (102)	288

Notes:

1. 6931 of the men who had two visits provided data about knowledge and attitudes relevant to maternal health outcomes

2. Only 3388 men gave responses about knowledge and attitudes relevant to child health outcomes on at least two visits because men visited for the first time early in the pregnancy did not answer questions related to child health on that visit

Table S3. Results from final multivariate models of associations between individual male characteristics and positive changes in knowledge and attitudes

Outcome/independent variables	Crude OR	Weighted OR	95% CIca
Know at least one danger sign in pregnancy			
Had enough food in last one week	2.50	2.50	1.29 – 4.85
Know 3 or more danger signs during pregnancy			
Age 30 years or less	1.84	1.84	1.28 – 2.65
Know at least one danger sign in childbirth			
Had enough food in last one week	3.18	3.18	1.64 – 6.19
Know 3 danger signs in childbirth			
No variables associated			
Believe women should reduce heavy work in pregnancy			
No variables associated			
Believe women should reduce heavy work by 3rd trimester			
No variables associated			
Ever discussed pregnancy & childbirth with spouse			
Live in urban site	5.34	5.34	1.63 – 17.51
Often discussed pregnancy & childbirth with spouse			
Age 30 years or less	0.62	0.54	0.35 – 0.84
Have only one wife	1.44	1.67	1.02 – 2.72
Mention poor hygiene as cause of childhood diarrhoea			
No variables associated			
Know to give increased fluids& continued feeding for diarrhoea			
No variables associated			
Would not give anti-diarrhoea medicine to child with diarrhoea			
Age 30 years or less	1.56	1.53	1.00 – 2.33
Has some formal education	0.36	0.36	0.15 – 0.84

OR=odds ratio; 95%CIca=cluster adjusted 95% confidence interval (catchment area as cluster)
Notes:
1. Only visited men who began with negative attitudes or lack of knowledge are included. Positive change is when the initial negative knowledge or attitude became positive on the later visit.
2. Variables included in initial saturated models: urban vs rural residence; sex of the household head; enough food in the house in the last one week; age 30 or below vs older; one wife vs more than one; any formal education; better paying job vs low paid or none
